# The Genomic Observatories Metadatabase (GeOMe): A new repository for field and sampling event metadata associated with genetic samples

**DOI:** 10.1371/journal.pbio.2002925

**Published:** 2017-08-03

**Authors:** John Deck, Michelle R. Gaither, Rodney Ewing, Christopher E. Bird, Neil Davies, Christopher Meyer, Cynthia Riginos, Robert J. Toonen, Eric D. Crandall

**Affiliations:** 1 Berkeley Natural History Museums, University of California, Berkeley, California, United States of America; 2 Hawaii Institute of Marine Biology, University of Hawaii, Kaneohe, Hawaii, United States of America; 3 Biocode, LLC, Junction City, Oregon, United States of America; 4 Texas A&M University, Corpus Christi, Texas, United States of America; 5 Gump South Pacific Research Station, University of California, Moorea, French Polynesia; 6 Berkeley Institute for Data Science, University of California, Berkeley, California, United States of America; 7 National Museum of Natural History, Smithsonian Institution, Washington, DC, United States of America; 8 University of Queensland, St Lucia, Queensland, Australia; 9 School of Natural Sciences, California State University, Monterey Bay, Marina, California, United States of America

## Abstract

The Genomic Observatories Metadatabase (GeOMe, http://www.geome-db.org/) is an open access repository for geographic and ecological metadata associated with biosamples and genetic data. Whereas public databases have served as vital repositories for nucleotide sequences, they do not accession all the metadata required for ecological or evolutionary analyses. GeOMe fills this need, providing a user-friendly, web-based interface for both data contributors and data recipients. The interface allows data contributors to create a customized yet standard-compliant spreadsheet that captures the temporal and geospatial context of each biosample. These metadata are then validated and permanently linked to archived genetic data stored in the National Center for Biotechnology Information’s (NCBI’s) Sequence Read Archive (SRA) via unique persistent identifiers. By linking ecologically and evolutionarily relevant metadata with publicly archived sequence data in a structured manner, GeOMe sets a gold standard for data management in biodiversity science.

## The missing metadata

Documenting patterns of global biodiversity and understanding how that diversity is generated and maintained are important steps towards mitigating the effects of anthropogenic stressors [1–3], whether local or global. Genetic data are key to this effort as these data can be used to: (a) identify cryptic diversity, (b) define population structure and associated management units, (c) identify hot spots of genetic diversity for the conservation of adaptive potential, (d) study the mechanisms driving patterns of biodiversity to identify regions of high evolutionary potential [4,5], and (e) monitor the flux of both intra- and interspecific genetic diversity at a particular site or within a particular region [6]. Whereas there have been several coordinated efforts to document patterns of species diversity (e.g., Global Biodiversity Information Facility [GBIF, http://www.gbif.org/; see Table 1 for acronym definitions], Ocean Biogeographic Information System [http://www.iobis.org/]), there have been fewer attempts to document and archive global patterns of genetic diversity. Notable efforts in this direction, however, include the Earth Microbiome Project [7,8] and Ocean Sampling Day [9], focusing on microbes, the Genomic Observatories Network (GO Network) of research sites focusing on entire ecosystems [10,11], and analyses of data archived in public repositories [12].

**Table 1:** Acronym definitions.

Category	Acronym	Name
**Databases**	Dryad	Dryad Digital Repository
	GBIF	Global Biodiversity Information Facility
	GeOMe	Genomic Observatories Metadatabase
	SRA	NCBI's Sequence Read Archive
**Organizations**	EMBL-EBI	European Bioinformatics Institute
	EMP	Earth Microbiome Project
	GO Network	Genomic Observatories Network
	GSC	Genomic Standards Consortium
	NCBI	US National Center for Biotechnology Information
	NSF	US National Science Foundation
	RCN	Research Coordination Network
	TDWG	Biodiversity Information Standards Organization, aka Taxonomic Databases Working Group
**Standards**	MIxS	GSC’s Minimum Information about any (x) marker Sequence
	RDF	Resource Description Framework
	DwC	Darwin Core, TDWG’s body of standards for sharing information about biological diversity
**File formats**	FASTA	Fast Alignment Search Tool-All
	FASTQ	Fast Alignment Search Tool-Quality
**Tools**	EZID	Tool for creating and managing globally-unique, long-term identifiers for data
	FIMS	Biocode Field Information Management System

While granting agencies and publishers enforce data accessibility and open access requirements for genetic data, they do not always require standardized metadata [13–15]. The public genetic repositories, such as NCBI and the European Bioinformatics Institute (EMBL-EBI), were established to store large volumes of sequence data. With vast capacity for storage and curation of genetic data, their role as repositories for the growing volume of genetic data is crucial; however, NCBI, for example, encourages but does not require the standardized metadata needed for ecological- or evolutionary-level analyses. Yet standards do exist for such metadata, notably thanks to the efforts of the Genomic Standards Consortium (GSC) [16] and the Biodiversity Information Standards Organization (known as “TDWG,” http://www.tdwg.org/). The GSC’s Minimum Information about any (x) marker Sequence (MIxS) standard [17] specifies a set of metadata standards for genetic data. Likewise, TDWG’s Darwin Core is a body of standards for describing and sharing biodiversity information [18]. However, neither NCBI nor EMBL-EBI currently enforces these standards or offers a portal for searching MIxS-compliant data. The problem is not only with the genetic repositories. The Dryad Digital Repository is an important resource that links data to their associated scientific publications and makes those data citable, yet Dryad does not enforce set standards or metadata requirements.

New databases and repositories that accommodate specific disciplines and subfields are coming online, e.g., http://reefgenomics.org/ [19], but there remains no central cross-disciplinary repository that enforces MIxS standards for sequence data and requires submission of the associated metadata describing the ecological and geographic context of source tissues. This “metadata gap” means that vital information about sampling events, such as sampling location, date, habitat, and organism life history, are rarely reported. Instead, most of this information is left unpublished, greatly diminishing the potential value (reuse) of the data [13,14,20].

## Filling the metadata gap: GeOMe

To fill the metadata gap for genetic sequence data, we have developed a web-based database and infrastructure to aid collaboration and the cross dissemination of published genetic data (http://geome-db.org/). GeOMe can be easily expanded as necessary to accommodate an increasing diversity of data from various research communities. Early development began as part of the Moorea Biocode Project (http://biocode.berkeley.edu/, Moore Foundation) and subsequently the National Science Foundation (NSF) Biological Science Collections Tracker project (http://biscicol.blogspot.com/). Development continued under a NSF Research Coordination Network (RCN) grant [16], which led to the establishment of the GO Network [10,11] as a joint initiative of GSC and the Group on Earth Observations Biodiversity Observation Network [21]. The resulting informatics stack (Biocode Commons) reached its current level of development under the auspices of another NSF RCN (the Diversity of the Indo-Pacific Network, http://diversityindopacific.net/) and is now being expanded for the broader scientific community as GeOMe.

**Fig 1:**
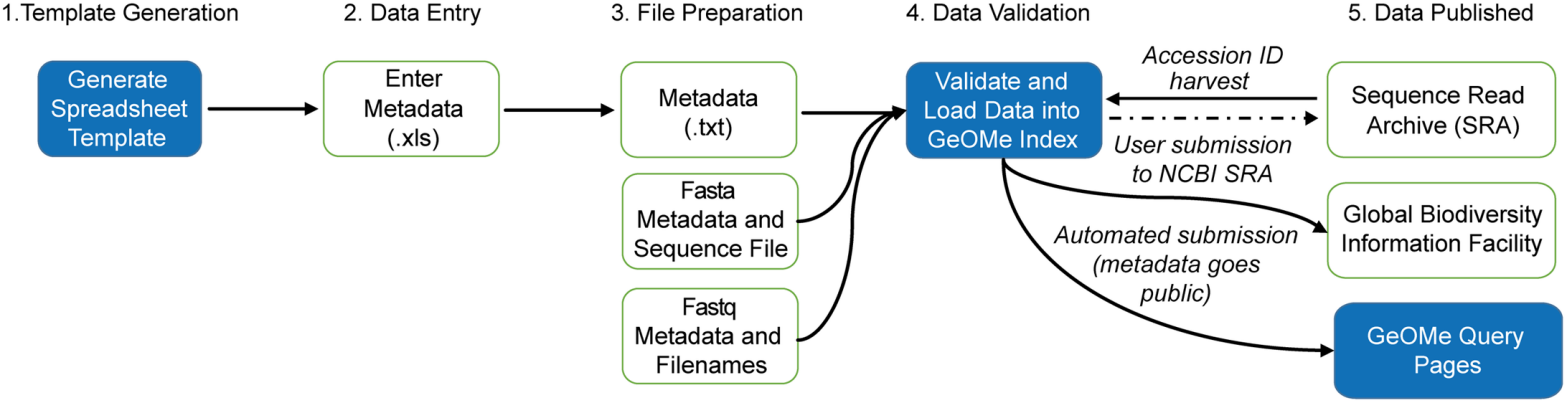
The Genomic Observatories Metadatabase (GeOMe) workflow. Steps in blue are those conducted within the Field Information Management System (FIMS) of GeOMe while those in white are independent of GeOMe.

The suite of tools provided through GeOMe provides a platform for investigators to publish standardized metadata that captures the temporal, environmental, geospatial, and even scholarly context for each sample and its derivative genetic data. GeOMe’s user-friendly, web-based interface allows users, from student and single investigator–driven projects to large scientific consortia, to customize metadata templates using the Biocode Field Information Management System (FIMS) [22]. Users select from a set of fields constructed from standard Darwin Core terms (http://rs.tdwg.org/dwc/) to create a metadata template that best reflects their needs and can be reused across multiple projects within or between labs (Fig 1). Data field options include hypotheses about the taxon (if an individual organism) or taxa in the sample (e.g., bacteria) and information on sampling habitat, life history (if an individual organism), details of sampling location and time, and publications deriving from the data. GeOMe provides a set of customizable project-level metadata validation rules, which ensures that metadata are compliant with both Darwin Core and MIxS standards (i.e., each sample has a unique identifier and required fields are provided). Thus, research communities can easily design their own templates and validation rules to describe, for example, an environmental sample used in metagenomics, tissues associated with transcriptomics, or an individual organism’s genomic sequence. Once the metadata template has been created, no internet connection is required for template editing until the data are uploaded, and therefore the system can be used in remote locations and with any personal computer that employs spreadsheet software (e.g., Microsoft Excel or comma-separated value [CSV] formats are supported).

The FIMS architecture (https://github.com/biocodellc) draws on community vocabularies (Darwin Core and MIxS) with terms stored internally as Uniform Resource Identifiers (URIs) and as specified by the Resource Description Framework (RDF) model. Most user-supplied data are stored as attributes of a core “sample” and are joined to either Sanger-based sequence data (including the marker name and actual sequence) or high throughput sequence data (storing metadata associated with sequence data stored on NCBI’s SRA). RDF-based attributes and class names for samples and sequences are then indexed in a document-store database (ElasticSearch, http://www.elastic.co/) for fast retrieval.

To submit data to GeOMe (Fig 1), contributors upload a tab-delimited text file together with a Fast Alignment Search Tool-All (FASTA) file (for Sanger sequence data) or a list of Fast Alignment Search Tool-Quality (FASTQ) file names (for high throughput sequence data, in which FASTQ files contain data from an individual sample). GeOMe then validates the dataset, checking to ensure that a set of minimum required fields are complete (following project-specific rules) and that sequence identifiers match metadata identifiers. When rules are violated, an informative and easy-to-interpret error message appears, prompting the user to fix the issue before proceeding. The contributor is also presented with a map of sampling localities to allow them to verify the geospatial information. Once validated, GeOMe assigns persistent, universally unique identifiers to each sample (EZID: California Digital Library; http://ezid.cdlib.org/), which are used for linking samples between GeOMe, NCBI, and other repositories. Sanger sequence data are stored as a text field within the database. For high throughput sequence data, GeOMe provides the data contributor with a completed batch metadata file for NCBI’s SRA and a SRA BioSample file to facilitate submission of the data to the NCBI SRA. Once the data are uploaded to the SRA, GeOMe harvests the NCBI accession numbers, thereby creating a direct link between the genetic data, the sample EZID, and associated metadata. To maximize open access, metadata are available under a Creative Commons Zero license (CC0) and are automatically pushed to GBIF using a dedicated Integrated Publishing Toolkit (IPT, http://www.gbif.org/ipt) installation [23]. Finally, users can choose to embargo their uploaded datasets from public view for a period of up to 2 years from the date of submission. While we encourage all users to make their data immediately public and CC0 on upload, we recognize that GeOMe is useful in preparing and processing research outputs and, consequently, data may not be ready for public release.

**Fig 2:**
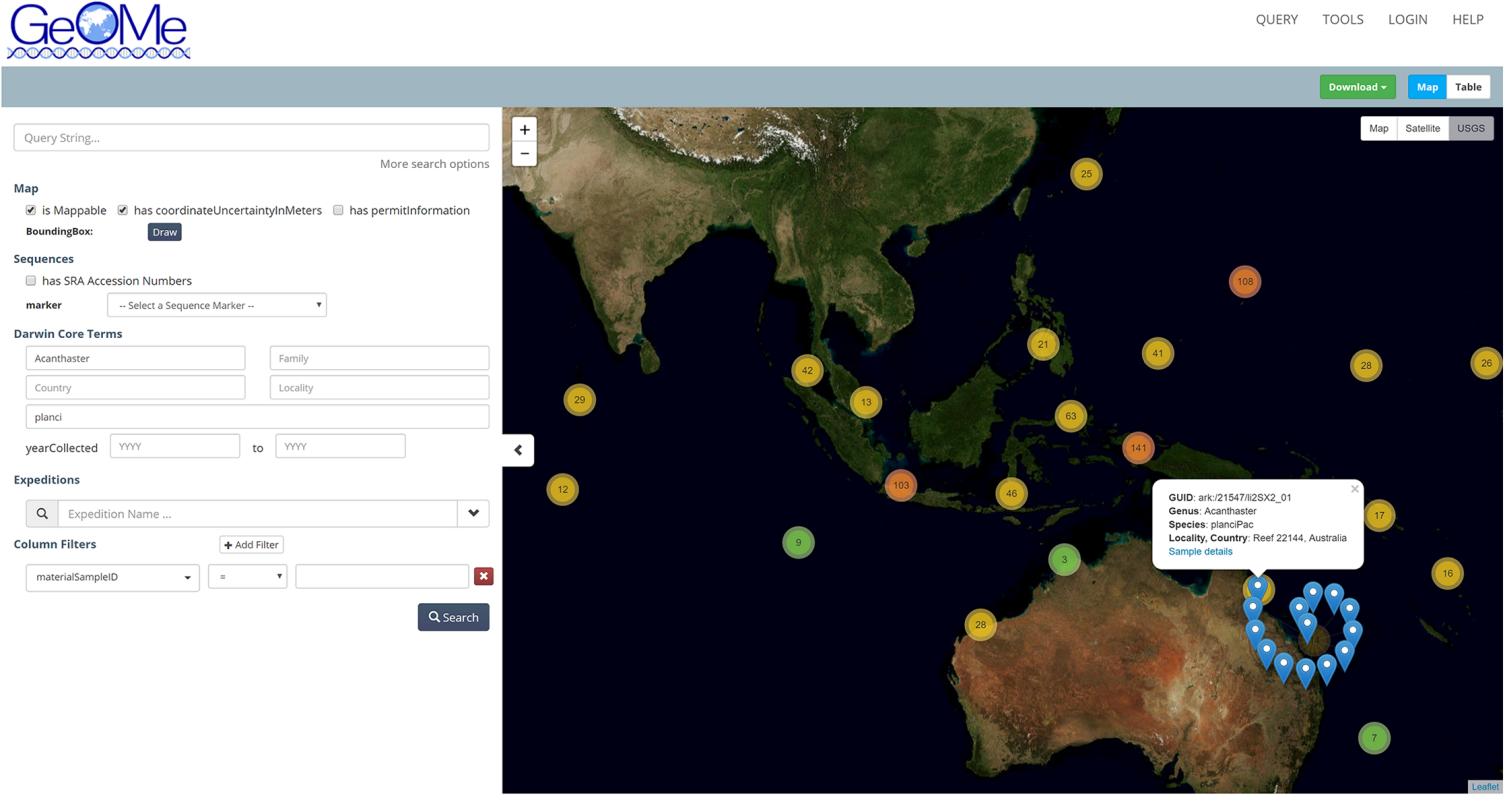
Screen shot of the Genomic Observatories Metadatabase (GeOMe) query system for *Acanthaster planci*, the crown of thorns sea star. Each number indicates the number of specimens in the database from that location. When a group of specimens is selected, distinct samples are visible as a spiral radiating from the chosen location, and individual records report summary information about each sample.

GeOMe is designed for flexibility and persistence using representational state transfer (REST) web services for communication between the database and the interface, while enabling potential third party applications to interact with services, as well. GeOMe’s web interface enables flexible searches based on any field and/or a geospatial bounding box (Fig 2). The GeOMe database may also be queried with a dedicated R package (geomedb; https://github.com/DIPnet/fimsR-access). GeOMe has also been designed so that it can be used in conjunction with the Biocode Laboratory Information Management System (LIMS; http://software.mooreabiocode.org) for the Geneious software platform (Biomatters, Incorporated). Sanger sequence data are available for download in FASTA format, while high throughput sequence data are provided as a list of SRA accession numbers. Associated metadata can then be downloaded in CSV and keyhole markup language (KML) formats. Already, the database contains metadata for >35,000 Sanger sequences across 233 species supplied from >50 participating laboratories. It has recently begun accepting metadata for high throughput FASTQ datasets. By using the FIMS architecture for metadata but continuing to store genetic sequence data at NCBI, we are helping to ensure long-term persistence of links between sequence data and its associated metadata while keeping the data searchable with NCBI’s Basic Local Alignment Search Tool (BLAST). We believe that this flexibility enables maximum integration with similar regional or discipline-specific data archival initiatives.

## Conclusion

A major challenge for biodiversity genomics research is the need to carry out physical sampling in the field (nucleotide sequences cannot be obtained remotely) and then to link biologically and ecologically important metadata with downstream data products, notably, published genetic sequences. No existing federated database provides this functionality. Yet, maintaining linkages among these data types is vital for data integration and analysis. Publicly archiving these metadata is essential to ensure scientific reproducibility and synthesis as well as to maximize potential reuse of sequence data as new techniques develop. Here, we provide a solution to the metadata gap: GeOMe. A bottom-up effort with buy-in from over 50 laboratories, our database is growing and adding new capacity while also setting the industry standard for metadata publication.

## Abbreviations

BLAST: Basic Local Alignment Search Tool
CC0: Creative Commons Zero license
CSV: comma-separated value
EMBL-EBI: European Bioinformatics Institute
FASTA: Fast Alignment Search Tool-All
FASTQ: Fast Alignment Search Tool-Quality
FIMS: Biocode Field Information Management System
GBIF: Global Biodiversity Information Facility
GeOMe: Genomic Observatories Metadatabase
GO Network: Genomic Observatories Network
GSC: Genomic Standards Consortium
IPT: Integrated Publishing Toolkit
KML: keyhole markup language
LIMS: Laboratory Information Management System
MIxS: GSC’s Minimum Information about any (x) marker Sequence
NCBI: US National Center for Biotechnology Information
NSF: US National Science Foundation
RCN: NSF Research Coordination Network
RDF: Resource Description Framework
REST: representational state transfer
SRA: NCBI’s Sequence Read Archive
TDWG: Biodiversity Information Standards Organization
URI: Uniform Resource Identifiers
